# Comparing traditional and AI-enhanced mentorship for leadership development in medicine

**DOI:** 10.1016/j.clinsp.2026.100956

**Published:** 2026-04-12

**Authors:** Ayla Mohtat, Fabio Ynoe Moraes, Alireza Mansouri, Ruth Gotian

**Affiliations:** a Penn State Health, Hershey, PA, USA; b Queen's University, Kingston, ON, Canada; c Weill Cornell Medicine, Anesthesiology, New York, NY, USA

## Introduction

Mentorship is a dynamic, reciprocal relationship and a foundational strategy for developing future leaders. In medicine, it is especially critical, fostering research productivity, career advancement, satisfaction, and leadership development for both mentors and mentees.^1^

Traditionally, mentorship involves structured or informal interactions ‒ ranging from clinical settings to virtual communication ‒ built on three pillars: i) *Longitudinal guidance*, ii) *Skill development*, and iii) *Network expansion*. However, increasing clinical demands challenge the consistency and depth of these interactions.

Meanwhile, the rise of Artificial Intelligence (AI) offers new tools to enhance mentorship efficiency and access but introduces ethical and interpersonal concerns that warrant careful consideration.

In this article, “leadership skills” refer to a combination of interpersonal, cognitive, and professional competencies ‒ such as confidence, adaptability, collaboration, and ethical decision-making ‒ that enable individuals to lead, communicate, and engage effectively.

### Traditional mentorship

Mentorship is traditionally practiced in form of apprenticeship models, defined as the relational dynamic between a more experienced clinician who guides the development of the less-experienced mentee. It usually takes on one of two mentorship models ‒ dyadic or non-dyadic. Dyadic mentorship is the most common form, consisting of one-on-one interactions.^2^^,^^3^ On the other hand, non-dyadic mentorship models – also known as group mentorship – are where two or more mentors and/or mentees are paired with each other.^2^ Regardless of the mentorship model, there is a “hidden curriculum” that is often recognized in traditional mentorship relationships. This refers to the implicit norms – also referred to as “power skills” – that mentees learn from their mentors and apply to their academic and personal life.^4^ These unspoken norms can be the decisive factor in an individual’s success and the development of their leadership skills. These can include social and professional expectations such as developing relationships with clinical team members and faculty, leadership and initiative, clear communication, email etiquette, and career path development, among others.

Along with mentors helping mentees navigate gray areas in academic professionalism, traditional mentorship can foster increased psychosocial support and human connection. Through engaged listening, providing positive and constructive feedback, and emotional reassurance during periods of academic or personal stress, mentees have been shown to feel valued, have an increased confidence, and a sense of belonging.^4^

### AI-Augmented mentorship

AI and other advanced technologies are being implemented rapidly in the current age, with advancements in diagnoses, drug discovery and development, and health monitoring, among others. Given these immense technological advancements, it is important to consider the potential culture shift surroundings mentor/mentee relationships-including both the opportunities and the challenges it can bring towards fostering initiative and clever decision-making.

In this context, AI-enhanced mentorship refers to a mentorship model that is mainly supported by AI to support, educate, and provide suggestions to promote the mentee’s development. These tools can be beneficial for several aspects of life and is available 24/7, which has rendered this mentorship model appealing to mentees, particularly with limited access or inconsistency with traditional mentors.

In addition to its full availability, AI poses several other advantages including providing mentees with grammatical feedback, decision-making support, and generation of ideas. Additionally, a main predictor of burnout among medical students is emotional exhaustion.^5^ highly due to the vast body of medical knowledge, which continues to expand. Students can find it difficult to not only keep up with their academic commitments, but also research initiatives, other curricular activities, and maintaining a work-life balance. AI software can be helpful in this domain, by summarizing information, developing practice questions, and explaining difficult concepts in methods that are personalized to individual learning styles.^6^ making information easier to understand and retain. It can also aid in scheduling, task prioritization, and other organizational duties.

**Fig. 1:**
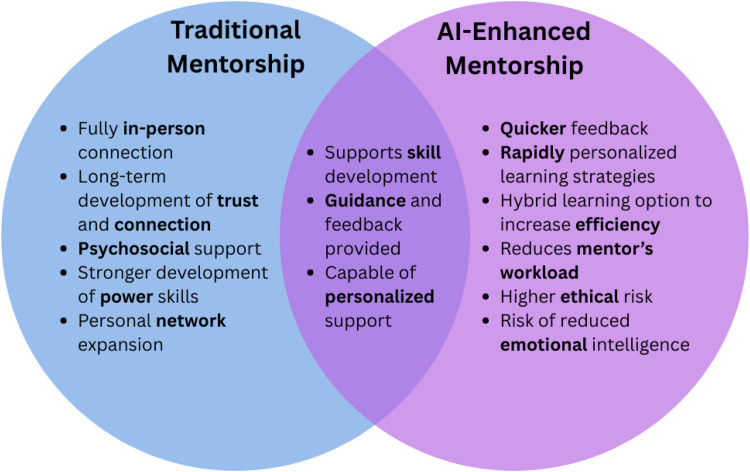
Traditional mentorship/ AI-enhanced mentorship.

Having established the three core pillars that ground mentorship, it is pertinent to examine to which extent AI-focused mentorship corresponds to these principals in comparison to traditional methods, as illustrated in Fig. 1. In terms of *longitudinal guidance*, AI’s help in tracking goals and monitoring progress over time can seem like a form of useful guidance. However, it could not replicate the human connection and evolving trust that characterizes traditional human mentorship-elements that are essential for building confidence and the interpersonal skills required to lead effectively. The second pillar, *skill development,* could initially present as a strong match for AI-enhanced mentorship, as many skills can be explained to mentees easily by AI, such as data analysis, and difficult medical concepts with easy-to-understand tutorials, and personalized study notes. However, as previously outlined, traditional mentorship contains a “hidden curriculum”.^4^ social norms developed in person, which AI cannot replicate. Finally, the last pillar, *network expansion*, could be supplemented by AI-enhanced mentorship by providing relevant contacts, events, and supporting one’s online presence (e.g., through CV refining, email optimization), however is not as substantial as the benefits provided with traditional mentorship, where one makes meaningful connections and initiates leadership.

Despite the various useful and time-efficient benefits AI provides, its guidance remains primarily technical. In contrast, meaningful mentorship, which promotes emotional intelligence and leadership, stems from acquiring power skills through real-life exchanges and clinical settings.

### Risks of AI-focused mentorship

Leveraging AI as a learning tool is beneficial, however can introduce challenges. Alongside its evident ethical concerns when it comes to misuse and plagiarism, it can also result in a loss of critical thinking and judgement. While AI can be helpful with summarization and explanation, it cannot teach ambiguity, resolve conflict, or navigate high-stress situations. As Messeri L. et al. have observed, many scientists who rely on AI as a source of understanding information may have an illusion of objectivity-believing they comprehend the full scope of a subject, when their understanding is much more limited.^7^ This can lead to surface-level thinking and can be detrimental to their critical thinking skills, which are essential to having the insight to lead others and communicate clearly.^8^

An additional challenge with AI-focused mentorship is the risk of misinformation.^9^ Studies have shown that since AI is anthropomorphized, and referred to with human adjectives, individuals put more trust into its responses, especially given its confident and well written statements. It becomes increasingly difficult for less experienced individuals to determine whether the statement is correct, causing individuals to believe faulty answers. Leadership, however, is grounded on integrity and discernment. It is essential for leaders to be knowledgeable on the topics they discuss, in order to be confident and effectively convey their message.

### Maintaining a balance

Given the respective strengths and limitations of both traditional and AI-focused mentorship, it is important to consider a balance between both mentorship models to maximise leadership-building potential. In the beginning stages of the mentoring relationships, it is important to determine appropriate integration of AI. While AI can assist with many tasks, human mentorship must be grounded on trust and accountability. Thus, with the ethical use of AI, as well as regular face-to-face meetings both online and in-person, mentorship can remain efficient while also maintaining depth and human connection.

In the age of AI, and with the demanding schedules of mentors in the medical field, mentors should focus on a leadership-focused strategy. This includes setting clear expectations, developing projects that cultivate critical thinking and problem-solve abilities, as well as communication skills, rather than simple task completion. This is because strong leaders’ main drivers are passion, and vision-they must be confident and passionate about the topic they are either speaking or writing about. In order to take initiative, they not only need to complete their work well, but also understand it and care about it deeply to authentically inspire others.

**Fig. 2:**
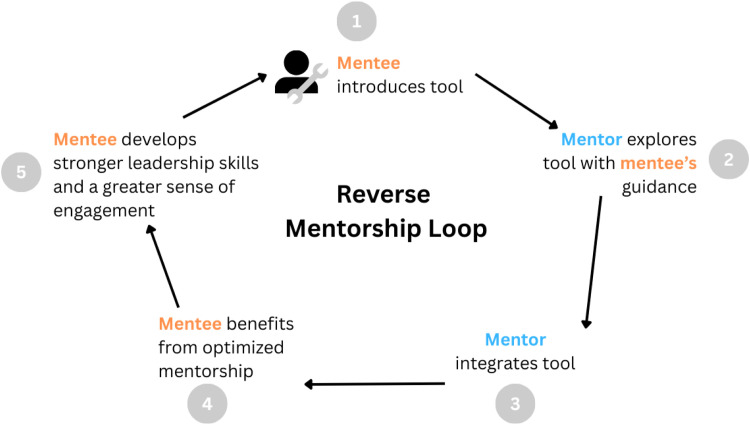
Reverse mentorship loop.

While mentors play a crucial role in providing guidance to mentees and building their network, as the medical field and technologies evolve, so do mentoring dynamics. As noted earlier, mentors in the medical field are often occupied with very demanding schedules, possibly lacking time to explore new developments in technology. Reverse mentorship is crucial in bridging this knowledge gap. First integrated by Jack Welch, the CEO of General Electric, who had the junior employees teach the senior executives about the internet, reverse mentorship consists of mentees guiding their mentor(s) in areas they may lack proficiency.^10^ as shown in Fig. 2. This concept deeply encourages initiative through all its stages-through the introduction pitch, as well as in guiding their mentor. It puts the mentee in the mentor’s shoes and allows them to further experience how to lead effectively.

## Conclusion

AI is undeniably transforming both the medical field and mentoring relationships, offering powerful and useful insights while simultaneously providing guidance. However, it is important to consider the benefits both AI-focused and traditional mentorship provides and implement both to optimize mentor-mentee relationships. Maintaining this balance is essential to foster leadership skills such as clear communication, clear judgement, and courage. This can be done both by using AI to enhance workflow and maintaining in-person and online meetings. It must be enforced that AI is not being used to replace the deeply personal and nuanced nature of mentorship. Thus, the most effective and successful mentorship will be those that integrate both AI’s benefits with the irreplaceable human elements of guidance and trust.

## Data availability

The datasets generated and/or analyzed during the current study are available from the corresponding author upon reasonable request.

## Declaration of competing interest

The authors declare no conflicts of interest.
